# Clinical evaluation of myocardial involvement in acute myopericarditis in young adults

**DOI:** 10.1186/s12872-017-0564-8

**Published:** 2017-05-22

**Authors:** Ersin Saricam, Yasemin Saglam, Tuncay Hazirolan

**Affiliations:** 1 Cag Hospital and Medicana International Ankara Hospital, Cardiology Clinic, Ankara, Turkey; 2 Department of Radiology, School of Medicine, Hacettepe University, Ankara, Turkey; 3 Present Address: Medicana International Ankara Hospital, Sogutozu District 2165 St. No: 6 Sogutozu, Ankara, Turkey

**Keywords:** Acute myopericarditis, Echocardiography, Myocardial focal echobright

## Abstract

**Background:**

Myocardial involvement in young adults has various causes. Acute myopericarditis is one of the myocardial involvements in young adults. It is easy to confuse with acute ST-elevation myocardial infarction because of the electrocardiographic features. This study aims to investigate a number of imaging techniques and clinical features for acute myopericarditis in young adults (<30 years of age).

**Methods:**

This retrospective study included 147 patients selected from the 2147 patients at the age of <30 with acute chest pain admitted into emergency service between 2010 and 2016. Of 147 patients, 77 patients were diagnosed with acute myopericarditis (group I) (between 18 and 30 aged) and 70 patients had ST-elevation myocardial infarction (group II). The echocardiographic pictures and information of the patients in both groups were rechecked in terms of impaired segmental wall-motion abnormalities, pericardial effusion, and additional features.

**Results:**

The patients in group I had focal echobright, which was defined as myocardial brightness in the left ventricle regions, especially in posterior and lateral wall. Focal echobright was observed in the 75 of 77 cases of acute myopericarditis in transthoracic echocardiogram. This sign was confirmed by cardiac magnetic resonance imaging. Focal echobright sensitivity was 95%; its specificity was 93%; its predictive was 95.2%. Pericardial effusion (83%) was observed in group I behind posterior wall. Its specificity was 81%; its sensitivity was 65%; predictivity was 73%.

**Conclusions:**

Pericardial effusion and myocardial focal echobright in echocardiography can be quite sensitive indicators for acute myopericarditis in young adults.

## Background

Myocardial involvement in young adults (<30 years age) has various causes other than atherosclerosis-related acute myocardial infarction. Among these may be drug abuse, congenital coronary abnormalities, coronary spasm, trauma, systemic vasculitis, and hematologic disease [1]. Acute myopericarditis, one of the myocardial involvements in young adults, manifests itself with chest pain, increased cardiac biomarkers, and ST-segment elevation, ST-T changes in electrocardiography [2–4]. Due to ST-segment elevation in electrocardiography, acute myopericarditis can mimic ST-segment elevation myocardial infarction and thus distinguishing between acute myopericarditis and ST-elevation myocardial infarction may prove difficult. Furthermore, some patients can undergo coronary angiography with a false diagnosis of acute myocardial infarction [5]. According to the ESC Position statement on myocarditis, for the diagnosis of myocarditis coronary angiography and endomyocardial (EMB) biopsy are required [6, 7]. If EMB endomyocardial biopsy is not possible, the diagnosis is called “suspected diagnosis of myocarditis” with or without associated pericarditis, and can be supported by Cardiovascular Magnetic Resonance (CMR) according to Lake-Louise criteria [8, 9]. However, CMR is not available in most centers in Turkey, while echocardiography is widely available. One of the most commonly used device in most centers, echocardiography is a practical tool. In this study, we investigated a number of imaging techniques (echocardiography, CMR) diagnostic assessments and clinical features with acute myopericarditis in young adults.

## Methods

This retrospective study included 147 patients selected from the 2147 patients at the age of <30 with acute chest pain admitted into emergency department (ED) between 2010 and 2016. Of the 147 patients, 77 patients were diagnosed with acute myopericarditis (between 18 and 30 ages) and 70 patients (between 20 and 30 ages) had ST-elevation myocardial infarction. All of the patients’ data (physical examination, electrocardiogram, echocardiographic picture, and blood analyzing with biochemical measurements) were re-analyzed. The acute myopericarditis diagnosis was made considering typical acute pericarditis electrocardiography features and increased cardiac troponin-I level. The echocardiographic pictures and information of the patients with acute myopericarditis diagnosis were rechecked in terms of impaired segmental wall-motion abnormalities, pericardial effusion, and additional features. The two echocardiography readers were blinded to the diagnosis so as not to have any biases in interpreting the images. Standard transthorasic echocardiography was performed in left lateral decubitus position with Vingmed System Five Advantage echocardiography device (General Electric, USA) 2.5 MHz transducer and two dimensional images (2-D), M-mode and Doppler. Depth in operation was 20 cm, dynamic range was 6. M-mode and 2-D images of left ventricle were taken based on the criteria of American Society of Echocardiography [10]. Three patients underwent CMR imaging within 72 h (see Fig. 2 for one patient’s CMR).

Twenty patients of group I (20/77) underwent coronary angiography, while all the patients in group II underwent coronary angiography (70/70). Echocardiography was performed for the patients in both groups.

The national guidelines do not require any approval of an ethic committee, for this is a retrospective study.

## Results

Fifty-five of the 77 patients with acute myopericarditis (group I) had previous upper respiratory tract infection histories. The patients in group I had pressure pain or sharp and pressure types (mixed). During physical examination, a pericardial friction rub was heard in 35 patients (35/77). In group I, 52 of the 77 patients had diffuse ST-elevation in all derivation; 19 patients had inferior (in lead D2, D3, aVR) ST-segment elevation. Six patients had lateral ST-segment elevation. Of the patients in the group II, 33 had inferior ST-segment elevation, 10 lateral ST-segment elevation, 27 anterior ST-segment elevation in electrocardiography.

All of the patients were monitored in terms of cardiac enzyme during follow-up (CK, CK- MB, and troponin). While cardiac troponin-I level increased up to 30 ng/ml in acute myopericarditis patients, in group II, it increased up to 90 ng/ml (Table 1).

**Table 1:** Differential diagnosis in acute myopericarditis

	Acute myopericarditis
ECG	ST concave elevation, PR depression except for aVR
History	Upper respiratory tract infection
Type of pain	Mixed
Cardiac MRI (T2)	Myocyte damage, edema, inflammation
Endo-myocardial biopsy (acute phase)	Interstitial edema, myocyte damage, lymphocytic infiltration
Coronary angiography	Normal
Echocardiography	Transient left ventricular wall thickening, pericardial effusion, tissue Doppler abnormality, detection of regional contractile and perfusion abnormalities in strain imaging/mapping and myocardial contrast echocardiography, myocardial heterogeneity
Therapy	Anti-inflammatory treatment

In group I, coronary angiography showed normal angiogram, while in group II patients had slow coronary artery (38/70), coronary atherosclerosis induced stenosis (10/70), drug abuse-induced coronary spasm (resolved by intracoronary nitroglycerin) (3/70), mad honey intoxication induced spasm (1/70), vasculitis-induced coronary involvement (5/70), trauma-induced myocardial contusion (2/70), anxieties-induced coronary spasm (2/70), spontan coronary dissection (4/70), coronary thrombosis (4/70), coronary abnormalities (2/70) (Table 2).

**Table 2:** The causes of the myocardial involvement in young adults (Group II) (<30 years age)

Patients in Group II	*n* = 70
Slow coronary artery	38
Coronary atherosclerosis	10
Vasculitis	5
Spontan coronary dissection	4
Coronary thrombosis	4
Drug abuse	3
Trauma	2
Anxieties induced coronary spasm	2
Coronary artery abnormalities	2
Mad honey intoxication	1

## Echocardiographic evaluation

Segmental-wall motion abnormalities were seen in the patients in both groups. Segmental-wall motion abnormalities in acute myopericarditis group were not compatible with electrocardiography and echocardiographic regions. For instance, acute myopericarditis cases with anterior ST-segment elevation had no impairment of apical and anterior segments, but posterior and lateral walls were affected. Segmental-wall motion abnormalities in ST-elevation myocardial infarction group were compatible with ST-segment.

Pericardial effusion in posterior wall behind (83%) was observed in group I. Its specificity was 81%; its sensitivity was 65%, and its predictivity was 73% (Fig. 3).

### Additional echocardiographic features, focal echobright, and CMR

**Fig. 1:**
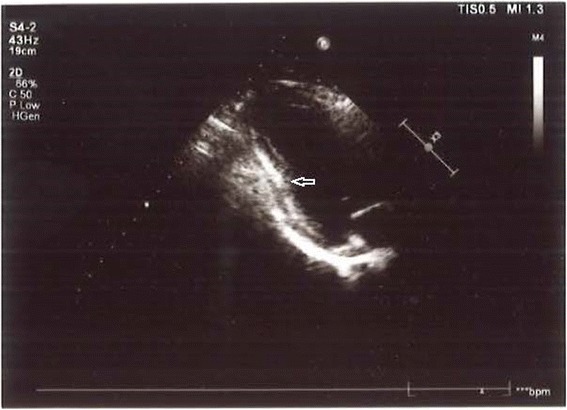
Focal echobright sign in posterolateral wall in echocardiography in acute myopericarditis (Patient 1)

**Fig. 2:**
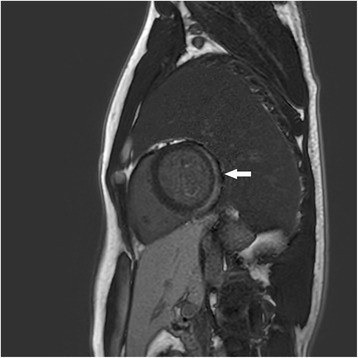
MRI imaging in the short axis. Posterior and posterolateral late gadolinium enhancement in the subepicardial region in with acute myopericarditis (Patient 1)

**Fig. 3:**
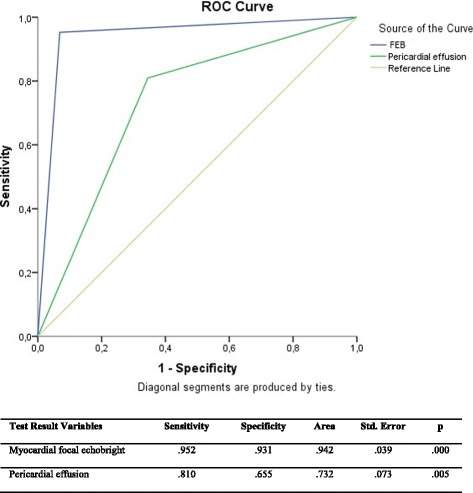
Test results for myocardial focal echobright and pericardial effusion

Most of the patients in group I (75/77) had myocardial brightness in the left ventricle regions, especially in posterior and lateral wall. We defined this appearance as focal echobright. It was most frequently seen in the posterolateral wall. Figure 1 shows the focal echobright in the posterolateral wall in echocardiographic appearance. However, there was no such appearance in the patients in group II. Three patients in group I underwent CMR, in which we saw a late gadolinium enhancement in the subepicardial region concordantly with echocardiographic focal echobright. This sign was confirmed by CMR. Figure 2 shows late gadolinium enhancement in the posterolateral wall in left ventricle short axis CMR. Focal echobright sensitivity was 95%, and specificity was 93%, and predictivity was 95.2% (Fig. 3).

In group I, the patients diagnosed with acute myopericarditis took indomethacin/ibuprofen for 3 weeks during non-steroidal therapy. These patients (excluding one patient) with acute myopericarditis were completely cured by rest and non-steroidal therapy. In two patients, an electrical problem developed (atrial extrasystol, temporary first atrioventricular block), which was resolved through medical therapy. One patient underwent temporary pacemaker implantation due to symptomatic temporary second-degree Mobitz type I (Wenckebach) AV block. One patient who had high troponin I (30 ng/ml) level died of cardiogenic shock. A definite myopericarditis diagnosis would have been made if an autopsy could have been performed; however, it was not possible as the relatives of the patients had no consent for the autopsy.

## Statistical analysis

The patient characteristics and outcomes for group I and group II were compared (Table 3). Mann-Whitney U Test was used to compare the variables. *P* value <0.05 was considered statistically significant.

**Table 3:** Patient characteristics

	Acute myopericarditis group (*n* = 77)	STEMI group (*n* = 70)
Age range	18–30	20–30
Male/Female	49/28	53/17
ECG abnormality	ST elevation, PR elevation	ST elevation
Upper respiratory tract infection history	yes	no
Pain type	mixt (sharp and pressure like)	typical
Pericardial friction rub	35/77	2/70
Pericardial effusion	83%	7 (10%)
Segmental wall motion abnormality -ECG	Incompatible	compatible
Myocardial heterogeneity	yes	none

## Discussion

Acute myopericarditis refers to the inflammation of visceral pericardium (epicardium) together with adjacent myocardium. The causes of acute myopericarditis are most frequently infectious (especially viral causes), immunological, caused by drug side-effects, malignancy, and radiation. Due to the acute electrocardiography appearance similar to ST-elevation myocardial infarction in EDs, differential diagnosis is very important [11]. In addition to acute chest pain, the most important electrocardiography finding of acute myopericarditis is ST-segment elevation [12].

There are several theories to explain the ST changes in acute myopericarditis. The first theory is myocardial inflammation resulting in mural thrombus and coronary artery embolisation. The second theory is the effect of catecholamine and vasoactive kinins causing spasm in acute phase period of viral infection in coronary arteries. The third is thrombus formation in coronary arteries due to arteritis and platelet activation [13].

Differential diagnosis in patients with no reciprocal ST-segment changes may be difficult. Differential characteristics of acute myopericarditis may be listed as the patient’s being younger than 40 years old, new viral infection findings, and electrocardiography changes occupying more than one vascular region (10). Electrocardiography provides additional information. While ST-segment in ST-elevation myocardial infarction appears as convex, ST-segment in acute myopericarditis is seen as concave. PR-segment depression in acute myopericarditis is observed except for aVR lead. Q-wave is rarely seen in acute myopericarditis. Early repolarization has also precordial ST-elevation, but cardiac enzymes are normal and ST-segment/T wave amplitude ratio is below 0.25 [14].

Coronary angiography is an invasive technique used to differentiate between acute myopericarditis and ST-elevation myocardial infarction. While coronary angiography in acute myopericarditis is normal, ST-elevation myocardial infarction case has serious lesions responsible for infarct. However, distinctive diagnosis between acute myopericarditis and ST-elevation myocardial infarction may sometimes be difficult. Khavandi et al. have reported a case of a 25-year-old man with acute streptococcal myopericarditis mimicking acute myocardial infarction [5] and have performed coronary angiography. Additionally, Salisbury et al. noted that the patients with acute coronary syndrome underwent coronary angiography in their health center and 16.8% of them were diagnosed with acute pericarditis [15].

In differential diagnosis, CMR may be used. It may play a significant role in avoiding invasive diagnosis [16]. It can noninvasively show myocardial tissue edema, myocyte impairment, and inflammation [17, 18]. Cardiovascular magnetic resonance criteria for myocarditis (Lake Louise Criteria) include regional myocardial edema, hyperemia in images acquired early after contrast injection, and inflammatory necrosis in images acquired late (>10 min) after contrast injection [19]. In a study comparing cardiac MRI and echocardiography, abnormal myocardial delayed enhancement was seen on cardiac MRI in 21 of 23 (91%) patients. Regional rather than global involvement was seen mainly in the inferolateral segments [20].

In endomyocardial biopsy, cardiomyocyte injury, myocardial inflammation (lymphocyte infiltration), and interstitial edema are seen in acute myopericarditis [21], whereas coagulation necrosis and neutrophilic infiltration are observed histologically in ST-elevation myocardial infarction [22].

Echocardiography studies in acute myopericarditis are limited in number. An increase in the left ventricular wall thickness in acute myopericarditis was shown in an echocardiographic and histo-pathological study conducted by Hiramitsu et al. The increase in the wall thickness was thought to be related to interstitial edema [23]. Lynch et al. evaluated mitral annular and velocity vector imaging in acute myopericarditis [24]. Afonso et al. stated that strain imaging and real- time myocardial contrast echocardiography supported the detection of regional contractile and perfusion abnormalities in acute myocarditis [25]. Similarly, Escher et al. suggested that speckle tracking echocardiography was a useful tool for evaluation acute myocarditis [26]. In addition, we observed a focal echobright sign in echocardiography in the assessment of acute myopericarditis. Since a focal echobright is present only in acute acute myopericarditis cases, this sign might be related to the acute phase of inflammation. Late gadolinium subepicardial enhancement in CMR supports this echocardiographic focal echobright. On the other hand, pericardial effusion was observed behind the posterior wall in acute myopericarditis [27]. We did not observe any pericardial tamponades. While the Lake Louise Criteria consider the presence of pericardial effusion as a supportive criterion only, other recent information has suggested that the assessment of pericardial effusion may improve the sensitivity of the CMR scan [28, 29].

## Limitations

Endomyocardial biopsy indicating interstisiel edema could not be performed.

## Conclusions

The causes of myocardial involvement in young adults are varied. Distinctive diagnosis of acute myopericarditis might sometimes prove difficult. Echocardiography is a non-invasive, rapidly accessible application which helps the clinician during the clinical evaluation of the patient in acute myopericarditis. Pericardial effusion and focal echobright can be quite sensitive findings in acute myopericarditis. Further research is required.

## Abbreviations

AV block: Atrioventricular block
CK- MB: Creatine kinase-MB
CK: Creatine kinase-MB
CMR: Cardiac magnetic resonance imaging
ESC: European Society of Cardiology
